# Fauchard

**Published:** 1852-10

**Authors:** 


*Fauchard.*—In another part of the present number of the Journal will be found an article over the signature of Fauchard, but in publishing it, we do not wish to be considered as endorsing all which the writer says. It is spiritedly written; a vein of sparkling humor runs through the whole article. It contains some excellent suggestions, and may do good. We would like to hear from Fauchard again. If we had a score or two of such men enlisted in the advancement of dental science, the evils to which he refers, would soon cease to exist.

